# Antioxidant Effects of Sheep Whey Protein on Endothelial Cells

**DOI:** 10.1155/2016/6585737

**Published:** 2016-03-31

**Authors:** Efthalia Kerasioti, Dimitrios Stagos, Vasiliki Georgatzi, Erinda Bregou, Alexandros Priftis, Ioannis Kafantaris, Dimitrios Kouretas

**Affiliations:** Department of Biochemistry and Biotechnology, University of Thessaly, Ploutonos 26 & Aiolou, 41221 Larissa, Greece

## Abstract

Excessive production of reactive oxygen species (ROS) may cause endothelial dysfunction and consequently vascular disease. In the present study, the possible protective effects of sheep whey protein (SWP) from tert-butyl hydroperoxide- (tBHP-) induced oxidative stress in endothelial cells (EA.hy926) were assessed using oxidative stress biomarkers. These oxidative stress biomarkers were glutathione (GSH) and ROS levels determined by flow cytometry. Moreover, thiobarbituric acid-reactive substances (TBARS), protein carbonyls (CARB), and oxidized glutathione (GSSG) were determined spectrophotometrically. The results showed that SWP at 0.78, 1.56, 3.12, and 6.24 mg of protein mL^−1^ increased GSH up to 141%, while it decreased GSSG to 46.7%, ROS to 58.5%, TBARS to 52.5%, and CARB to 49.0%. In conclusion, the present study demonstrated for the first time that SWP protected endothelial cells from oxidative stress. Thus, SWP may be used for developing food supplements or biofunctional foods to attenuate vascular disturbances associated with oxidative stress.

## 1. Introduction

Free radicals such as reactive oxygen species (ROS) can be generated in a wide variety of chemical and biological systems. ROS play an important role in body's immune response [1], redox regulation of gene transcription [2], and cell signaling [1]. On the other hand, the ensuing cascade of ROS can result in cellular damage including apoptosis, protein oxidation, DNA modification, and lipid peroxidation [3]. Under normal conditions ROS are controlled by antioxidant systems. When there is a disturbance between the prooxidant and antioxidant balance in favor of the former that leads to oxidative stress which can cause damage to all molecular targets [1], a range of antioxidants are active in the body including enzymatic and nonenzymatic antioxidants [4]. Antioxidant enzymes include superoxide dismutase (SOD), catalase (CAT), and glutathione peroxidase (GPX) [4]. Nonenzymatic antioxidants include vitamin A, vitamin C, vitamin E, flavonoids, glutathione (GSH), uric acid, and bilirubin [5].

The endothelium lines the entire vascular system and is composed of a monolayer of endothelial cells. Endothelial cell structure and functional integrity are important in the maintenance of the vessel wall and circulatory function. In addition to its role as a selective permeability barrier, endothelial cells are dynamic and are capable of conducting a variety of metabolic and synthetic functions and regulating homeostasis, immune, and inflammatory responses [6]. Endothelial cell injury or dysfunction is a hallmark of many pathologic conditions including atherosclerosis and thrombosis [6]. Excessive production of ROS may exceed the capacity of antioxidant mechanisms, thus contributing to vascular disease by induction of endothelial dysfunction through several pathways [6]. Endothelial dysfunction is considered largely as endothelial activation, which may eventually contribute to arterial disease [6]. Inflammatory cytokines, growth factors, and the interaction of the endothelium with leukocytes may induce ROS signaling in endothelial cells. Moreover, interaction between ROS and NO may cause a vicious circle leading to more endothelial activation and inflammation [6]. In addition, superoxide dismutase may use superoxide radical (O_2_^∙−^) for producing hydrogen peroxide which can diffuse to the endothelial cells and damage proteins through reaction with cysteine groups [7]. Thus, continuous ROS signaling in endothelial cells can cause loss of integrity, progression to senescence, and detachment into the circulation [8].

Thus, there is a great interest for natural sources of antioxidants in order to enhance antioxidant mechanisms and protect the organism from the harmful effects of oxidative stress. For example, whey protein is a widely consumed supplement that is considered to increase the antioxidant defense [9, 10]. Whey protein is a by-product of cheese manufacturing, but it is used as a functional food with nutritional applications [11, 12]. The main components of whey include beta-lactoglobulin, alpha-lactalbumin, bovine serum albumin, lactoferrin, immunoglobulins, lactoperoxidase enzymes, glycomacropeptides, and lactose [13]. Some of these components act as antioxidants. For example, alpha-lactalbumin can chelate iron and thus result in the reduction of oxidative stress [14]. Moreover, whey protein has a high content in the sulphur-containing amino acids cysteine and methionine that enhance antioxidant mechanisms through intracellular conversion to glutathione [11].

In our previous studies, we have shown that a cake containing sheep whey protein (SWP) had antioxidant and anti-inflammatory activities in subjects submitted to intense exercise [9, 15]. We have also shown that SWP exerted antioxidant effects on C2C12 muscle cells [16]. The aim of the present study was to examine the possible protective effects of SWP against tert-butyl hydroperoxide- (tBHP-) induced oxidative stress in EA.hy926 endothelial cells.

## 2. Materials and Methods

### 2.1. Chemicals, Reagents, and Culture Medium

Dulbecco's modified Eagle's medium (DMEM), fetal bovine serum (FBS), phosphate buffered saline (PBS), and L-glutamine and trypsin were purchased from Gibco (Grand Island, NY). Tert-butyl hydroperoxide (tBHP), 2,4-dinitrophenylhydrazine (DNPH), urea, oxidized glutathione (GSSG), nicotinamide adenine di-nucleotide phosphate (NADPH), 5,5′-dithiobis (2-nitrobenzoic acid) (DTNB), 2-vinyl pyridine, glutathione reductase, ethyl acetate, Bradford reagent, mercury orange, and 2,7-dichlorofluorescein diacetate (DCF-DA) were obtained from Sigma-Aldrich (St. Louis, MO, USA). Trichloroacetic acid (TCA), sodium hydroxide (NaOH), 2-thiobarbituric acid (TBA), and ethanol were purchased from Merck (Darmstadt, Germany). Cell proliferation kit II (XTT) was purchased from Roche Diagnostics (Mannheim, Germany).

### 2.2. Cell Culture

EA.hy926 endothelial cells were cultured as described previously in tissue culture flasks at 37°C in 5% CO_2_ [17]. The medium used was DMEM, containing 10% (v/v) FBS, 2 mM L-glutamine, 100 units mL^−1^ of penicillin, and 100 units mL^−1^ of streptomycin (Gibco, UK).

### 2.3. Cell Viability Assay

Cell viability was assessed using the XTT assay kit (Roche, Germany) as described previously [17]. Briefly, EA.hy926 cells were subcultured into a 96-well plate with 1 × 10^4^ cells per well in DMEM medium. After 24 h of incubation, the cells were treated with increasing concentrations of SWP (0.78, 1.56, 3.12, and 6.24 mg of protein mL^−1^) in serum-free DMEM medium for 24 h or tBHP (0.15, 0.3, 0.6, and 1.2 mM) for 1 h. Then, following manufacturer's instructions absorbance was measured at 450 nm and also at 690 nm as a reference wavelength in a Bio-Tek ELx800 microplate reader (Winooski, VT, USA). Cell cultures in DMEM serum-free medium were used as a negative control. The absorbance of each SWP concentration alone in DMEM serum-free medium and XTT test solution was also tested at 450 nm. The absorbance values shown by the proteins alone were subtracted from those derived from EA.hy926 cell treated with proteins. Data were calculated as percentage of inhibition by the following formula:

(1) $$\begin{array}{c} inhibition\left(\;\%\;\right)=\left[\;\frac{\left({O.D.}_{control}-{O.D.}_{sample}\right)}{{O.D.}_{control}}\;\right]\times 100, \end{array}$$

where O.D._control_ and O.D._sample_ indicated the optical density of the negative control and the tested compounds, respectively. All samples were measured in triplicate and at least in three independent experiments.

### 2.4. Determination of Conditions for the Treatment of EA.hy926 Cells with tBHP

In order to find out the appropriate conditions (i.e., dose, incubation time) at which tBHP-induced oxidative stress in EA.hy926 cells, the cells were seeded in 25 cm^2^ culture flasks, and when cell confluency was 70–80% incubated with tBHP for 1/2 or 1 h at 0.15 and 0.3 mM. Then, oxidative stress markers (i.e., ROS and GSH levels) were evaluated using flow cytometry for assessing oxidative stress induction.

### 2.5. Treatment of EA.hy926 Cells with SWP

EA.hy926 cells were seeded in 25 cm^2^ culture flasks for GSH and ROS determination and 75 cm^2^ culture flasks for TBARS, protein carbonyls, and GSSG determination and were incubated for 24 h at 37°C in 5% CO_2_. Then, at a cell confluency of 70–80%, the medium was removed and replaced with serum-free medium containing SWP at different concentrations (0–6.24 mg of protein mL^−1^), followed by incubation for 24 h. The untreated cells were considered as controls. After incubation, SWP was removed and tBHP (0.3 mM) was added for 1 h. Then, the cells were trypsinized, collected, and centrifuged twice at 300 g for 10 minutes at 5°C. Each centrifugation was followed by supernatant dismissal and resuspension of cellular pellet in PBS. After the last centrifugation the cellular suspension was used for the measurement of oxidative stress markers, namely, GSH, ROS, TBARS, protein carbonyls, and GSSG.

### 2.6. Flow Cytometric Analysis of GSH and ROS Levels

The intracellular GSH and ROS levels were assessed by flow cytometry using mercury orange and DCF-DA, respectively, as described previously [17]. In particular, the fluorescent mercury orange binds directly to GSH, while DCF-DA within cells is deacetylated by esterases and further converted to fluorescent DCF by oxidative action of ROS. A 400 *μ*M stock solution of mercury orange was made up in acetone and stored at 4°C, while a fresh 400 *μ*M stock solution of DCF-DA was prepared in methanol. To assess the GSH and ROS levels, the cells were resuspended in PBS at 1 × 10^6^ cells per mL and incubated in the presence of mercury orange (40 *μ*M) or DCF-DA (10 *μ*M) in the dark at 37°C for 30 min. Then, the cells were washed, resuspended in PBS, and submitted to flow cytometric analysis using a FACScan flow cytometer (Becton Dickinson, NJ, USA) with excitation and emission at 488 and 530 nm for ROS and at 488 and 580 nm for GSH. Also, forward angle and right angle light scattering showing the cells size and cell internal complexity, respectively, were measured. Cells were analyzed at a flow rate of 1000 events per second. Analyses were performed on 10000 cells per sample and fluorescence intensities were measured on a logarithmic scale of four decades of log of fluorescence. Data were analyzed by using BD Cell Quest software (Becton Dickinson). Each experiment was repeated at least three times.

### 2.7. Assessment of GSSG Levels

For the assessment of GSSG levels, cellular suspension was homogenized by sonication on ice. The resulting lysate was then centrifuged at 10,000 ×g for 10 min at 4°C. Afterwards, GSSG was measured in the supernatant according to the method of Reddy et al. [18]. Briefly, 50 *μ*L of supernatant was neutralized to pH 7.0–7.5 with NaOH. Then, 5 *μ*L of 2-vinyl pyridine (1 : 100 diluted) was added and the samples were incubated at room temperature for 2 h. Ten *μ*L of the sample treated with 2-vinyl pyridine was mixed with 600 *μ*L of 143 mM sodium phosphate (6.3 mM EDTA, pH 7.5), 100 *μ*L of 3 mM NADPH, 100 *μ*L of 10 mM DTNB, and 189 *μ*L of H_2_O. The samples were incubated for 10 min at room temperature. After the addition of 1 *μ*L of glutathione reductase, the change in absorbance at 412 nm was read for 3 min. The assay requires more than 2–4 *μ*g absolute amount of protein in the test sample. Total protein in cellular suspension was assayed using a Bradford reagent from Sigma-Aldrich. GSSG concentration was calculated using a standard sample containing 75 *μ*L of 10 *μ*mol L^−1^ oxidized glutathione.

### 2.8. Assessment of TBARS Levels

For the assessment of TBARS levels, cellular suspension was homogenized by sonication on ice. Then, TBARS were measured in the resulting homogenate spectrophotometrically as previously described [16]. 400 *μ*L of cellular suspension or 400 *μ*L of PBS for blank was mixed with 500 *μ*L of 35% TCA and 500 *μ*L of trishydroxymethylaminomethane hydrochloride (Tris-HCl) (200 mM, pH 7.4) and incubated for 10 min at room temperature. Afterwards, 1 mL of 2 M Na_2_SO_4_ and 55 mM TBA solution was added and the samples were incubated at 95°C for 45 min. The samples were cooled on ice for 5 min and were vortexed after adding 1 mL of 70% TCA. Then, the samples were centrifuged at 15,000 g for 3 min and the absorbance of the supernatant was read at 530 nm. The assay requires more than 30 *μ*g absolute amount of protein in the test sample. Total protein in cellular suspension was assayed using a Bradford reagent from Sigma-Aldrich. Calculation of TBARS concentration was based on the molar extinction coefficient of malondialdehyde (MDA).

### 2.9. Assessment of Protein Carbonyl Levels

For the assessment of protein carbonyl levels, cellular suspension was homogenized by sonication on ice. Then, protein carbonyls were measured in the homogenate spectrophotometrically as previously described [17]. In this assay, 200 *μ*L of 20% TCA was added to 200 *μ*L of cellular suspension and this mixture was incubated in an ice bath for 15 min and centrifuged at 15,000 g for 5 min at 4°C. The supernatant was discarded and 500 *μ*L of DNPH [in 2.5 N hydrochloride (HCL)] for the sample or 500 *μ*L 2.5 N HCL for the blank was added in the pellet. The samples were incubated in the dark for 1 h, with intermittent vortexing every 15 min and were centrifuged at 15,000 g for 5 min at 4°C. The supernatant was discarded and 1 mL of 10% TCA was added, vortexed, and centrifuged at 15,000 g for 5 min at 4°C. The supernatant was discarded and 1 mL of ethanol-ethyl acetate (1 : 1 v/v) was added, vortexed, and centrifuged at 15,000 g for 5 min at 4°C. This washing step was repeated twice. The supernatant was discarded and 1 mL of 5 M urea (pH 2.3) was added, vortexed, and incubated at 37°C for 15 min. The samples were centrifuged at 15,000 g for 3 min at 4°C and the absorbance was read at 375 nm. The assay requires more than 30 *μ*g absolute amount of protein in the test sample. Total protein in cellular suspension was assayed using a Bradford reagent from Sigma-Aldrich. Calculation of protein carbonyl concentration was based on the molar extinction coefficient of DNPH.

### 2.10. Statistical Analysis

Data were analyzed by one-way ANOVA followed by Tukey's test for multiple pairwise comparisons. The level of statistical significance was set at *P* < 0.05. For all statistical analyses SPSS, version 13.0 (SPSS Inc., Chicago, IL), was used. Data are presented as mean ± SEM.

## 3. Results

### 3.1. Determination of the Cytotoxic Activity of SWP and tBHP

**Figure 1:**
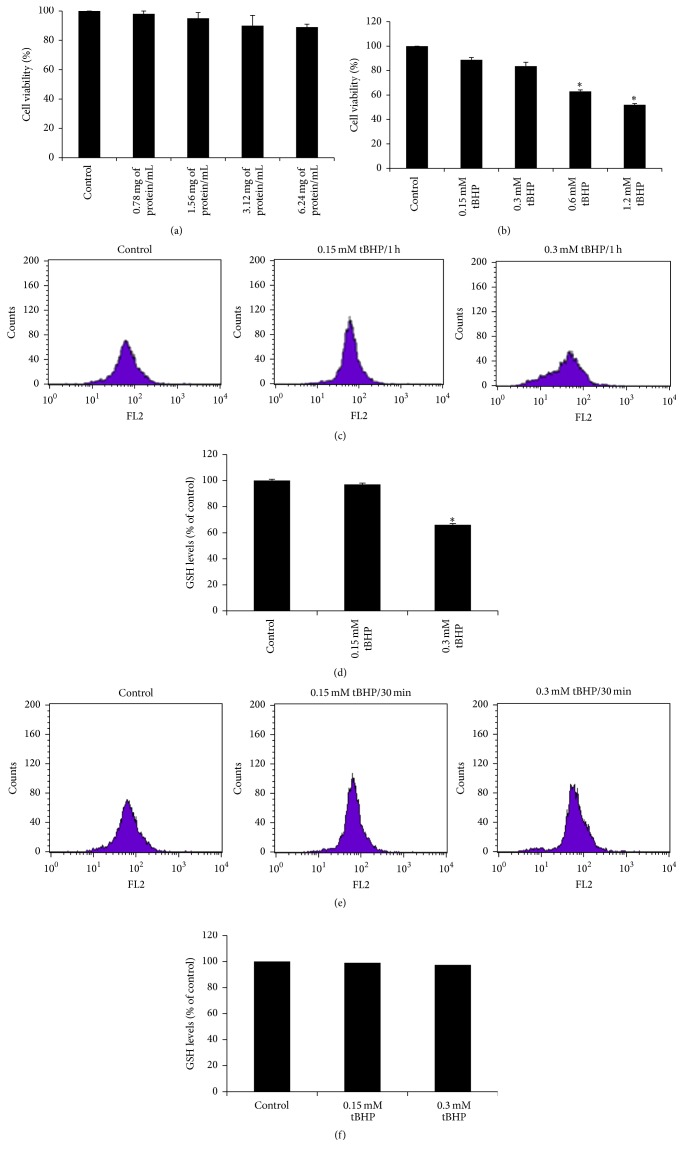
(a) Effects of whey protein on viability of EA.hy926 cells presented as % of control (untreated cells). (b) Effects of tBHP on viability of EA.hy926 cells presented as % of control (untreated cells). (c) The histogram of cell counts versus fluorescence of 10,000 cells analyzed by flow cytometer for the detection of GSH in EA.hy926 cells treated with tBHP at 0.15 and 0.3 mM for 1 h. FL2 represented the detection of fluorescence using 488 and 580 nm as the excitation and emission wavelength, respectively. (d) GSH levels in EA.hy926 cells treated with tBHP at 0.15 and 0.3 mM for 1 h, presented as % of control. (e) The histogram of cell counts versus fluorescence of 10,000 cells analyzed by flow cytometer for the detection of GSH in EA.hy926 cells treated with tBHP at 0.15 and 0.3 mM for 1/2 h. (f) GSH levels in EA.hy926 cells treated with tBHP at 0.15 and 0.3 mM for 1/2 h, presented as % of control. All values are presented as the mean ± SEM of 3 experiments (*n* = 9 for cell viability assay; *n* = 3 for GSH assay). ^*∗*^Statistically significant compared to tBHP alone (*P* < 0.05).

In the present study, the SWP was examined at concentrations (0.78, 1.56, 3.12, and 6.24 mg protein/mL) that exhibited antioxidant activity* in vitro* [16]. The cytotoxic activity of SWP in EA.hy926 cells was examined using the XTT assay. The results showed that SWP had no cytotoxic effect at the examined concentrations (Figure 1(a)). Regarding tBHP, the results showed that there was no cytotoxicity at concentrations below 0.6 mM (Figure 1(b)).

### 3.2. Determination of the Conditions for the Treatment of EA.hy926 Cells with tBHP

**Figure 2:**
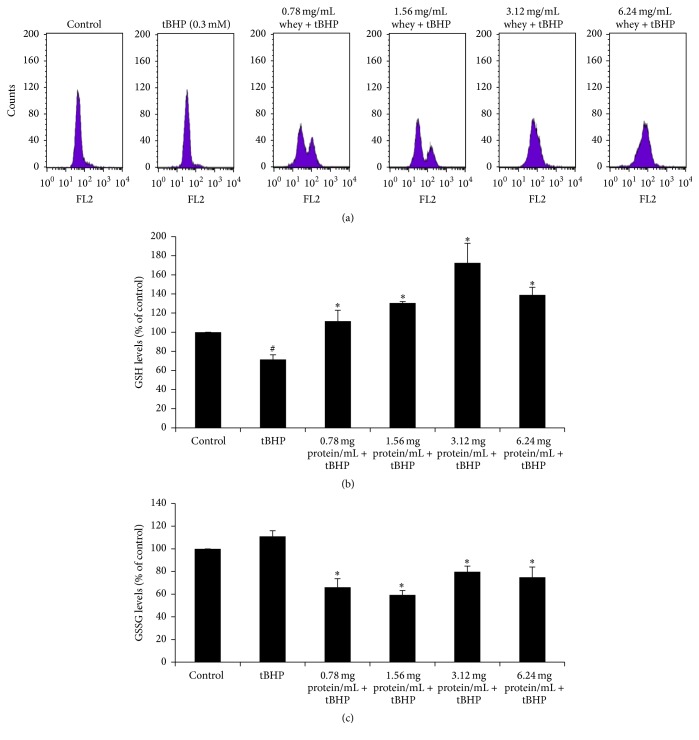
Effects of whey protein on GSH and GSSG levels in EA.hy926 cells. (a) The histogram of cell counts versus fluorescence of 10,000 cells analyzed by flow cytometer for the detection of GSH. FL2 represented the detection of fluorescence using 488 and 580 nm as the excitation and emission wavelength, respectively. (b) GSH levels in EA.hy926 cells presented as % of control. (c) GSSG levels as evaluated by spectrophotometer. Cells were studied under three conditions: under normal conditions (control), under treatment with tBHP (0.3 mM) for 1 h, and under the combination of whey protein (0.78–6.24 mg of protein mL^−1^) for 24 h and tBHP (0.3 mM) for 1 h. All values are presented as the mean ± SEM of 3 experiments (*n* = 3). ^*∗*^Statistically significant compared to tBHP alone (*P* < 0.05). ^#^Statistically significant compared to control (*P* < 0.05).

In a previous study, we have found that tBHP-induced oxidative stress in mouse C2C12 myoblastoma cells at 0.3 mM after 1/2 h incubation time. Based on these results, for finding the appropriate conditions (i.e., incubation time and dose) for the treatment of EA.hy926 cells with tBHP, the following methodology was followed. At first, as mentioned above, tBHP's effects on viability of EA.hy926 cells were examined, so as the noncytotoxic concentrations to be used. The results showed that there was no cytotoxicity at concentrations below 0.6 mM of tBHP (Figure 1(b)). Then, noncytotoxic concentrations (i.e., 0.15 and 0.3 mM) of tBHP were used at two different incubation times, 1/2 and 1 h. At these incubation times, the effect of tBHP at different doses on GSH and ROS levels was assessed, so as to find out the appropriate concentration at which tBHP-induced oxidative stress. The results showed that there was a tBHP-induced decrease in GSH levels at 0.3 mM after 1 h of incubation (Figures 1(c), 1(d), 1(e), and 1(f)). For this reason, these conditions were selected for tBHP treatment. Moreover, although tBHP at 0.3 mM did not increase ROS levels (Figures 2(a) and 2(b)), it increased lipid peroxidation and protein oxidation (Figures 4(a) and 4(b), resp.).

### 3.3. Effects of Sheep Whey Protein on GSH and GSSG Levels in EA.hy926 Cells

For assessing the effects of SWP on GSH, ROS, and TBARS levels in EA.hy926 cells, noncytotoxic concentrations (0.78–6.24 mg of protein mL^−1^) were used (Figure 1).

The GSH levels were evaluated by flow cytometry using mercury orange for staining. Histograms demonstrating the cell counts versus fluorescence of mercury orange are shown in Figure 2(a). The mean fluorescent intensity was evaluated using the BD Cell Quest software and the values are presented as percentage of the control (untreated cells) (Figure 2(b)). tBHP treatment decreased significantly GSH levels by 28.6% compared to controls (Figure 2(b)). However, treatment of EA.hy926 cells with SWP, at concentrations of 0.78, 1.56, 3.12, and 6.24 mg of protein mL^−1^, before tBHP administration increased GSH levels by 56.2%, 82.6%, 141%, and 95.5%, respectively, compared to tBHP treatment alone (Figure 2(b)). Although there was an increase in GSH levels as SWP concentration increased, there were no statistically significant differences between GSH values at different SWP concentrations (Figure 2(b)).

The results showed that treatment of EA.hy924 cells with tBHP had no significant effect on GSSG levels compared to control. However, pretreatment with SWP at concentrations of 0.78, 1.56, 3.12, and 6.24 mg of protein mL^−1^ decreased GSSG levels by 40.5, 46.7, 28.1, and 32.5%, respectively, compared to tBHP treatment alone (Figure 2(c)). Similar to GSH assay, SWP-induced decrease in GSSG levels was not dose dependent (Figure 2(c)).

### 3.4. Effects of Sheep Whey Protein on ROS Levels in EA.hy926 Cells

**Figure 3:**
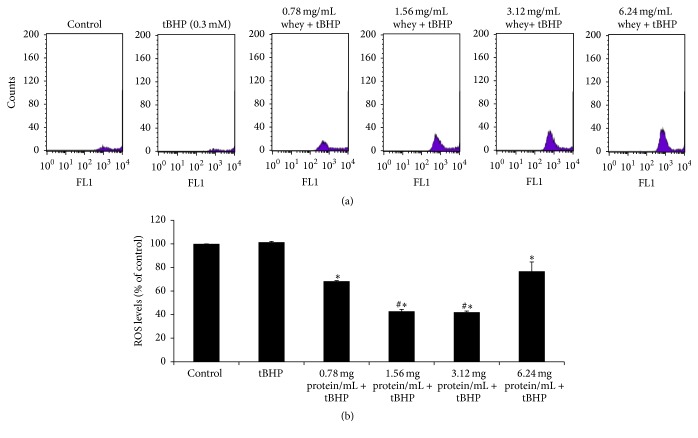
Effects of whey protein on ROS levels in EA.hy926 cells. (a) The histogram of cell counts versus fluorescence of 10,000 cells analyzed by flow cytometer for the detection of ROS. FL1 represented the detection of fluorescence using 488 and 530 nm as the excitation and emission wavelength, respectively. (b) ROS levels in EA.hy926 cells presented as % of control. Cells were studied under three conditions: under normal conditions (control), under treatment with tBHP (0.3 mM) for 1 h, and under the combination of whey protein (0.78–6.24 mg of protein mL^−1^) for 24 h and tBHP (0.3 mM) for 1 h. All values are presented as the mean ± SEM of 3 experiments (*n* = 3). ^*∗*^Statistically significant compared to tBHP alone (*P* < 0.05). ^#^Statistically significant compared to 0.78 mg of protein mL^−1^ (*P* < 0.05).

ROS levels were evaluated by flow cytometry using DCF-DA for staining. Histograms demonstrating the cell counts versus fluorescence of DCF-DA are shown in Figure 3(a). The mean fluorescence intensity values were evaluated using the BD Cell Quest software and are expressed as percentage of the control (untreated cells) (Figure 3(b)). The administration of tBHP did not affect ROS levels compared to control. However, treatment of EA.hy926 cells with SWP at concentrations of 0.78, 1.56, 3.12, and 6.24 mg of protein mL^−1^ before tBHP administration decreased significantly ROS levels by 32.6, 57.8, 58.5, and 24.4%, respectively, compared to tBHP treatment alone (Figure 3(b)). In this assay, ROS values at 1.56 and 3.12 mg of protein mL^−1^ were significantly lower compared to 0.78 mg of protein mL^−1^, indicating a dose dependent effect (Figure 3(b)).

### 3.5. Effects of Sheep Whey Protein on TBARS Levels in EA.hy926 Cells

**Figure 4:**
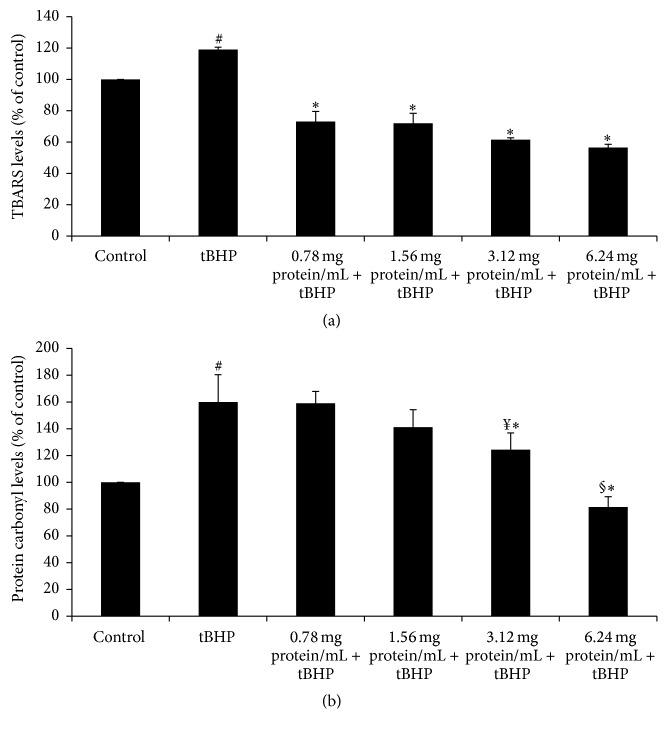
Effects of whey protein on (a) TBARS levels and (b) protein carbonyl levels as evaluated by spectrophotometer presented as % of control. Cells were studied under three conditions: under normal conditions (control), under treatment with tBHP (0.3 mM) for 1 h, and under the combination of whey protein (0.78–6.24 mg of protein mL^−1^) for 24 h and tBHP (0.3 mM) for 1 h. All values are presented as the mean ± SEM of 3 experiments (*n* = 3). ^*∗*^Statistically significant compared to tBHP alone (*P* < 0.05). ^#^Statistically significant compared to control (*P* < 0.05). ^¥^Statistically significant compared to 0.78 mg of protein mL^−1^ (*P* < 0.05). ^§^Statistically significant compared to 1.56 mg of protein mL^−1^ (*P* < 0.05).

The results showed that tBHP treatment increased significantly TBARS levels by 19.0% compared to control. Treatment of cells with SWP at 0.78–6.4 mg of protein mL^−1^ before tBHP administration led to a significant decrease in TBARS levels by 38.7, 39.4, 48.0, and 52.5%, respectively, compared to tBHP treatment alone (Figure 4(a)). Although there was a decrease in TBARS levels as SWP concentration increased, TBARS values were not significantly different among the different SWP concentrations (Figure 4(a)).

### 3.6. Effects of Sheep Whey Protein on Protein Carbonyl Levels in EA.hy926 Cells

Protein carbonyl levels were increased significantly by 60.0% after tBHP treatment compared to control. However, pretreatment of EA.hy926 cells with SWP at concentrations of 3.12 mg of protein mL^−1^ and 6.24 mg of protein mL^−1^ decreased significantly protein carbonyl levels by 22.0 and 49.0%, respectively, compared to tBHP treatment alone (Figure 4(b)). Moreover, there were significant differences in the protein carbonyl levels between 0.78 and 3.12 mg of protein mL^−1^ concentrations as well as between 1.56 and 6.24 mg of protein mL^−1^ concentrations suggesting a dose dependent effect of SWP (Figure 4(b)).

## 4. Discussion

An imbalance between ROS and antioxidants can lead to oxidative stress which causes lipid peroxidation, protein oxidation, and DNA damage, leading to several diseases [19]. All aerobic organisms including human have antioxidant mechanisms that protect against oxidative damage and repair damaged molecules. However, the natural antioxidant mechanisms may be insufficient and the supplementation with natural antioxidants through diet is of great interest. Such a natural product is whey protein, a by-product of cheese manufacturing, that is considered a functional food with a number of health benefits [11]. We have shown previously that SWP possesses antioxidant activity [9, 16]. In the present study, we investigated the protective effects of SWP against tBHP-induced oxidative stress in EA.hy926 endothelial cells.

For this purpose, the effects of SWP on GSH levels in EA.hy926 cells were examined. GSH is the most abundant antioxidant in aerobic cells, present in micromolar (*μ*M) concentrations in bodily fluids and in millimolar (mM) concentrations in tissues [20]. Because of the cysteine residue, GSH is readily oxidized nonenzymatically to glutathione disulfide (GSSG) by electrophilic substances (e.g., ROS) resulting in their scavenging [5, 21]. The GSSG efflux from cells contributes to a net loss of intracellular GSH [5]. Thus, the decrease of GSH:GSSG ratio is considered an indicator of oxidative stress [22]. EA.hy926 cell treatment with tBHP decreased GSH levels and increased GSSG levels. It has been reported that tBHP oxidizes GSH through the activity of glutathione peroxidase (GP_x_), thus leading to increased levels of GSSG [23]. However, pretreatment of cells with SWP before tBHP administration led to an increase in GSH levels and a decrease in GSSG levels compared to tBHP alone treatment. These results were consistent with those of other studies. For example, O'Keeffe and FitzGerald [24] have reported that incubation of human umbilical vein endothelial cells (HUVECs) with hydrolysate fractions of whey protein resulted in an increase in cellular glutathione by about 130%. In one of our previous studies, we have also shown that treatment of C2C12 muscle cells with increasing concentrations of SWP (0.78–6.24 mg of protein mL^−1^) before tBHP treatment increased GSH levels by 138% [16] and decreased GSSG levels by 31% (unpublished data) compared to tBHP treatment alone. Xu et al. [25] demonstrated that treatment of C2C12 cells with 0.5 mg mL^−1^ whey protein, under the influence of hydrogen peroxide (H_2_O_2_), increased GSH levels by 341% compared to H_2_O_2_ alone treatment. In another study, administration of 0.1, 1, and 10 mg mL^−1^ of whey protein, before ethanol exposure, increased GSH levels by 20.0%, 43.0%, and 98.0%, respectively, in the pheochromocytoma cell line PC12 [12]. The whey protein-induced increase in GSH levels is due probably to the contribution of cysteine residues that aid in the synthesis of GSH [13]. Furthermore, whey protein has been shown to induce the synthesis of GP_x_ eliminating hydroperoxides by oxidizing GSH to GSSG, which in turn is reduced to GSH by glutathione reductase (GR) [25].

Moreover, the effects of SWP on TBARS levels, a marker of lipid peroxidation, were examined. Treatment of EA.hy926 cells with tBHP increased significantly TBARS levels. It has been proposed that tBHP reacts with Fe^2+^ leading to the formation of tert-butyl hydroperoxide (tBO^∙^) radicals leading to lipid peroxidation [23, 26, 27]. The treatment of EA.hy926 cells with SWP decreased tBHP-induced increase of TBARS levels. Interestingly, in a previous study, we have found that pretreatment of C2C12 muscle cells with SWP (0.78–6.24 mg of protein mL^−1^) decreased tBHP-induced increase of TBARS levels up to 25.5% [16]. Moreover, we have shown that a cake containing SWP decreased plasma TBARS levels in athletes after intense exercise [9]. In another study, diabetic rats supplemented with whey protein exhibited a significant decrease in the level of malondialdehyde (MDA) levels, a marker of lipid peroxidation [28]. Moreover, Xu et al. [25] showed that in C2C12 muscle cells whey protein at 0.5 mg mL^−1^ is inhibited by 67% hydrogen peroxide-induced increase of MDA levels.

Furthermore, SWP treatment of EA.hy926 cells resulted in inhibition of tBHP-induced increase in protein oxidation, as shown by reduction in protein carbonyls. This effect is important, since oxidative stress-induced carbonylation of proteins leads to the loss of their physiological function [29]. It is believed that there is an association between lipid and protein oxidation [30]. For example, tBHP has been suggested to lead to the formation of tBO^∙^ radicals that in turn lead to protein oxidation either directly by attacking the amino acyl side chains or indirectly by leading to lipid peroxidation [26]. Thus, the SWP-induced decrease in lipid peroxidation may also account, at least in part, for inhibiting tBHP-induced increase in protein oxidation. Moreover, Haraguchi et al. [31] have shown that whey protein precluded increases in muscle protein carbonyl content in exercised and sedentary animals.

Intriguingly, tBHP treatment did not affect ROS levels. As we have suggested previously, it seems that although tBHP produces free radicals, their “free form” cannot be observed [17]. This may be attributed to the reaction of free radicals with other molecules in the cells. For instance, the decrease in GSH and the increase in lipid peroxidation and protein oxidation after tBHP treatment suggested that free radicals may react with GSH, lipids, and proteins, respectively. However, treatment of EA.hy926 cells with SWP before tBHP administration led to a decrease in ROS levels up to 58.5% compared to tBHP alone treatment. In one of our previous studies, we have also found that pretreatment of C2C12 muscle cells with SWP (0.78–6.24 mg of protein mL^−1^) decreased ROS levels to 41.3% [16]. Moreover, in another study, whey protein isolate (pWPI) and whey protein native hydrolysates (nWPI) at 2 mg mL^−1^ inhibited H_2_O_2_-induced ROS formation by 76.0% and 32.5%, respectively, in human colonic adenocarcinoma (Caco-2) cell line [32]. Likewise, whey protein has been shown to decrease significantly ROS levels in diabetic rats [28]. The decrease in ROS levels may be explained by the SWP-induced increase in antioxidant defense mechanisms such as GSH. Interestingly, recent clinical studies have shown that a whey protein formulation reduced by almost twofold inflammatory oxidative damage (IOD) levels [33] and improved vascular functions [33, 34].

Although SWP treatment enhanced antioxidant capacity of EA.hy926 cells by either increasing antioxidant mechanisms or reducing ROS levels and oxidative stress-induced damage, these effects were not always dose dependent. Thus, SWP exhibited dose dependent decrease in ROS and CARB levels but SWP-induced increase in GSH or decrease in GSSG and TBARS levels was not dose dependent. This may be explained by the different mechanisms through which SWP affects each of the tested oxidative stress markers, and so its potency differs among the different assays. It seems that in GSH, GSSG, and TBARS assays, SWP's activity has already reached a plateau at the concentrations used and for this reason a dose dependent effect was not observed. Namely, if lower than the tested concentrations were used, it may also be a dose dependent effect in these assays.

In conclusion, the present study showed that SWP was effective to protect endothelial cells from oxidative stress-induced damage. SWP exerted its protective activity against oxidative stress, by increasing GSH levels and decreasing GSSG, lipid peroxidation, protein oxidation, and ROS levels in EA.hy926 cells. It should also be mentioned that SWP concentrations (i.e., from 0.78 to 6.24 mg/mL) used were not cytotoxic. Moreover, in a previous study we have shown that at these concentrations SWP exhibited strong free radical scavenging activity and reducing power and enhanced the antioxidant capacity in mouse myoblastoma C2C12 cells [16]. Also, these concentrations are assimilated to the concentrations found in food. For example, the highest concentration of SWP was 6.24 mg/mL in the cell culture medium. Taken into account that the total plasma volume in human organism is about 3.5 L, then the concentration of 6.24 mg/mL of SWP would be achieved theoretically if about 20 g of SWP is consumed. This amount is within the range of the recommended intake doses of whey protein. Thus, since these whey protein concentrations can be found in blood, then the endothelial cells are possible to be exposed to them, since they are the main cells of the blood vessel walls. Thus, the findings of the present study suggest that SWP may be used as food supplement to attenuate vascular disturbances associated with oxidative stress.
